# Characterisation of sleep in intensive care using 24-hour polysomnography: an observational study

**DOI:** 10.1186/cc12565

**Published:** 2013-03-18

**Authors:** Rosalind Elliott, Sharon McKinley, Peter Cistulli, Mary Fien

**Affiliations:** 1 Faculty of Health, University of Technology Sydney, 235-253 Jones Street, Broadway 2007, New South Wales, Australia; 2 Intensive care unit, Royal North Shore Hospital, Northern Sydney Local Health District, Reserve Road, St Leonards 2065, New South Wales, Australia; 3 Faculty of Medicine, University of Sydney, Fisher Road, Camperdown 2006 New South Wales, Australia; 4 Centre for Sleep Health & Research, Royal North Shore Hospital, Reserve Road, St Leonards 2065, New South Wales, Australia; 5 Musculoskeletal Network, Agency for Clinical Innovation, 67 Albert Avenue, Chatswood 2067, New South Wales, Australia

## Abstract

**Introduction:**

Many intensive care patients experience sleep disruption potentially related to noise, light and treatment interventions. The purpose of this study was to characterise, in terms of quantity and quality, the sleep of intensive care patients, taking into account the impact of environmental factors.

**Methods:**

This observational study was conducted in the adult ICU of a tertiary referral hospital in Australia, enrolling 57 patients. Polysomnography (PSG) was performed over a 24-hour period to assess the quantity (total sleep time: hh:mm) and quality (percentage per stage, duration of sleep episode) of patients' sleep while in ICU. Rechtschaffen and Kales criteria were used to categorise sleep. Interrater checks were performed. Sound pressure and illuminance levels and care events were simultaneously recorded. Patients reported on their sleep quality in ICU using the Richards Campbell Sleep Questionnaire and the Sleep in Intensive Care Questionnaire. Data were summarised using frequencies and proportions or measures of central tendency and dispersion as appropriate and Cohen's Kappa statistic was used for interrater reliability of the sleep data analysis.

**Results:**

Patients' median total sleep time was 05:00 (IQR: 02:52 to 07:14). The majority of sleep was stage 1 and 2 (medians: 19 and 73%) with scant slow wave and REM sleep. The median duration of sleep without waking was 00:03. Sound levels were high (mean Leq 53.95 dB(A) during the day and 50.20 dB(A) at night) and illuminance levels were appropriate at night (median <2 lux) but low during the day (median: 74.20 lux). There was a median 1.7 care events/h. Patients' mean self-reported sleep quality was poor. Interrater reliability of sleep staging was highest for slow wave sleep and lowest for stage 1 sleep.

**Conclusions:**

The quantity and quality of sleep in intensive care patients are poor and may be related to noise, critical illness itself and treatment events that disturb sleep. The study highlights the challenge of quantifying sleep in the critical care setting and the need for alternative methods of measuring sleep. The results suggest that a sound reduction program is required and other interventions to improve clinical practices to promote sleep in intensive care patients.

**Trial registration:**

Australian New Zealand clinical trial registry (http://www.anzctr.org.au/): ACTRN12610000688088.

## Introduction

The role of good quality sleep in health is well known [1,2]. However, many critically ill patients treated in the intensive care unit (ICU) experience sleep disruption [3-6]. Intensive care patients may experience normal or near-normal total sleep time (TST) but have been reported to have reduced slow wave (SW) and rapid eye movement (REM) sleep, largely because of frequent arousals. The factors causing sleep disruption in intensive care patients are not fully understood; many extrinsic and intrinsic factors have been proposed, such as noise [7], light, inflammatory mediators [6], sedative and opioid medications [4] and mechanical ventilator settings [8]. Polysomnography (PSG) recording is supported by reports from ICU patients on their inability to sleep well in ICU [9,10]. Patients often attribute disturbances to their sleep to noise levels [10].

Twenty-four hour PSG studies in ICU have been few and there are no recent studies; there have been fewer studies still examining the prevalence of sleep disruptive factors simultaneously with PSG or investigations of ICU patient perceptions of the quality of sleep and sleep disruptions in conjunction with PSG recording. The primary aim of this observational study was to provide a current assessment of the quality and quantity of critically ill patients' sleep while they were treated in ICU. The secondary aims were to explore the prevalence of the main factors that potentially affect sleep in ICU, that is the environmental sound pressure and illuminance levels ICU patients are exposed to, and the frequency of treatment and care patients receive. We also assessed self-reported sleep quality while patients were treated in ICU and the hospital ward and patients' perceptions of sleep disruptions in ICU.

## **Materials and methods**

### *Study setting and sample*

This study was conducted in a 36-bed adult general ICU in a 600-bed hospital in Sydney, Australia. The ICU provided specialty services such as cardiac, spinal, burns and renal and was a closed unit with an accredited intensivist responsible for the management of all patients. Ward rounds were conducted by the intensivist twice a day when treatment goals/plans were reviewed, for example sedation medication prescriptions were adjusted according to the individual patient needs (routinely, calm and interactive). The registered nurse (RN) to patient ratio was 1:1 for mechanically ventilated patients and 1:2 for patients requiring high dependency care. There was no distinct area for the care of high dependency level patients; mechanically ventilated patients were cared for alongside patients of lower acuity. The RN performed all the nursing care for the patient. The main practices associated with sleep promotion during the time in which the study was conducted were offering a night-time hypnotic (that is, temazepam) and earplugs/eye shades to the patients and dimming the main lights at night.

Patients were included if aged >16 years and likely to be treated in ICU for >24 hours and able to give informed consent on their own behalf. The ability to provide consent was assessed by the following means: i) subjective advice by the bedside nurse; cognition was discussed including orientation to time and place and ability to follow simple instructions; ii) the patient was approached and asked to state their name (or mouth the words); if the patient was able to perform this instruction a further check was made; iii) the patient's understanding and ability to follow instructions was checked by asking them to nod when the correct colour card was held up from a selection of three. Exclusion criteria included a history of sleep disorders, psychiatric illness requiring medication, a known diagnosis of dementia or central neurological impairment confirmed by radiological scan. Human Research Ethics Committee approval for the study was provided by the Health Service and the University of Technology Sydney. Patients provided informed consent with written confirmation by their next of kin in cases where the patient was unable to sign the consent form. Data were collected from January to December, 2009 and September 2010 to April 2011. Screening for eligibility was performed on weekdays.

### *Data collection*

On enrolment, patients rated their sleep quality prior to hospitalization using a 1 to 10 scale (10 = excellent) and patients or their proxy completed the Insomnia Severity Index (ISI) [11]. The ISI comprises seven items based on the symptoms and severity of sleep disturbance; each item is scored from 0 (not at all) to 4 (extremely). The total ISI score ranges from 0 to 28 with a cutoff for clinical insomnia of 15. Concurrent validity with a sleep diary has been reported at r = 0.65 [11].

Patients were monitored for one 24-hour period using a portable PSG device, either PS-2™ (Compumedics, Melbourne, Australia) or ALICE LE™ (Philips Respironics, Amsterdam, Netherlands). Recording began and finished between 1000 and 1700 hours. Electroencephalograph (EEG) (O1/M2, C4/M1), electromyograph (EMG), electrooculograph (EOG) (right and left) and electrocardiograph (ECG) (lead II) were recorded. Patients' skin was prepared according to standard techniques. Gold cup EEG electrodes were placed at O1/M2 and C4/M1 according to the International 10-20 System [12]. Two EOG electrodes were used for right and left eye movements. The EMG electrodes were located over the right and left masseter (facial) muscles. Electrode application was performed by the authors (RE and MF) who were both trained in the technique. Electrode impedance was maintained <9,000 ohms. Visual checks were performed hourly and electrodes were replaced if impedance values approached 9,000 ohms or when the patient was disturbed for routine repositioning.

Sound and illuminance levels were recorded simultaneously with PSG using the integrated sound pressure level meter (model 2250, Brüel and Kjaer™, Nærum, Denmark) and illuminance level meter (T-10, Konica Minolta™, Osaka, Japan). Continuous equivalent sound pressure levels (Leq) in 'A' weighted decibels and peak sound pressure levels (Lpeak) in 'C' weighted decibels were logged every second. More detailed information about the protocol for sound pressure level recording and configuration of patient rooms is reported elsewhere [13]. Illuminance level (in lux) was recorded, using a sensor placed close to the patient's head, once per minute. The bedside nurse was requested to log an event whenever the patient received treatment or care using a specially designed Microsoft Access™ (Microsoft, Redmond, WA, USA) form listing a number of events on a computer located within reach. The event log contained the following items: clinical assessment; tracheal suctioning; pressure area care; physiotherapy; mouth/eye care; blood test (sampling); wash; non-invasive blood pressure; eating and drinking; dressing; pain; line insertion; X-ray; clinical crisis; agitation/anxiety/confusion; electrode replacement and other (for example placing an extra blanket on the patient).

On completion of PSG recording patients rated their previous night's sleep using the Richards Campbell Sleep Questionnaire (RCSQ) [14]. The RCSQ contains five 100mm visual analogue scales (VAS): sleep depth, latency, awakenings, time awake and quality of sleep (higher scores indicate better sleep). The RCSQ was pilot tested in a medical ICU [15] and validated with overnight PSG in medical ICU patients [14]. In our study, patients who were unable to write were assisted; the patient used their current communication strategy to indicate where the investigator should mark the VAS.

One to two nights after transfer to the ward, patients rated their sleep on the ward using the RCSQ and reported on sleep-disturbing factors in ICU using the Sleep in Intensive Care Questionnaire (SICQ) [10]. The SICQ was developed to determine the perceived effect of the ICU environment on sleep. It contains seven questions (rated 1 to 10), including overall sleep quality at home, sources of perceived sleep disruption and sources of noise.

Demographic and clinical data were collected from the patient's record. The Acute Physiology and Chronic Health Evaluation (APACHE) III modified diagnostic codes [16] were used to classify diagnoses. The APACHE II severity of illness score on admission and the Modified Sequential Organ Failure Assessment (SOFA) [17] at the time of enrolment were calculated to assess severity of illness.

### *Data analysis*

Polysomnography recordings were scored manually in 30-second epochs, by three qualified sleep technologists using standard Rechtschaffen and Kales [18] (R and K) criteria. TST was defined as the time spent in all sleep stages. The percentage of time in each sleep stage and sleep during the day (0600 to 2100 hours) was calculated. Arousals were defined using American Academy of Sleep Medicine criteria [19] and the number per hour of sleep (arousal index) reported. Morphine equivalent doses of opioid medications [20] and midazolam equivalent doses of benzodiazepine [21] were calculated to summarise the doses of opioid and sedative medications administered.

The software packages PASW™ (version 18; SPSS Inc, Chicago, IL, USA) and Microsoft Excel (2007) were used to analyse the data. Means and medians were used to describe continuous data and frequencies and percentages were used for categorical data. Interrater reliability for scoring the PSG data by sleep technologists was performed on 16 (30%) recordings using Cohen's Kappa statistic. The Mann-Whitney U test was used to detect differences between the TST and arousal indices for patients who received mechanical ventilation during PSG and those who did not and to compare arousal indices for patients who received benzodiazepine medications or propofol and those who did not. The correlation between arousal indices and peak sounds (>80 dB(A)) was explored using Pearson's r. Wilcoxon signed ranks test was used to compare patients' self-reported quality of sleep in ICU and the ward.

## Results

### *Sample characteristics*

**Figure 1:**
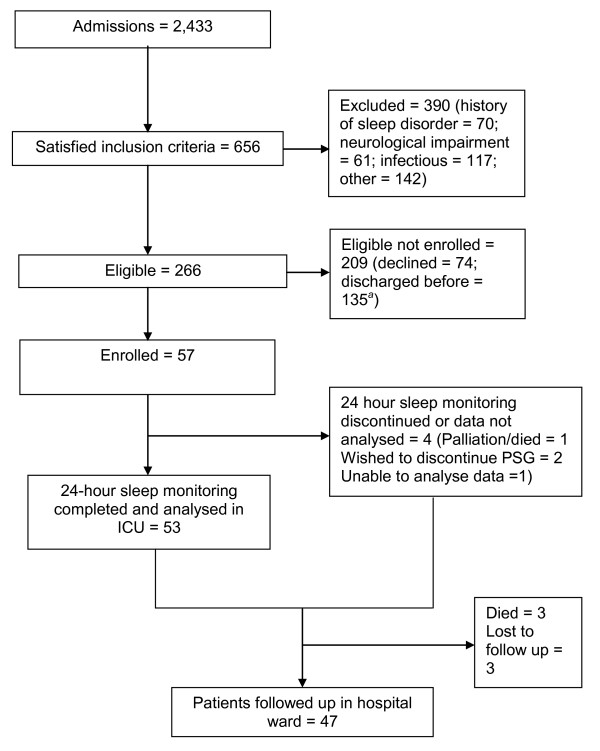
****Prevalence of eligible patients****. Number of patients admitted to ICU during the time period in which the study was conducted, eligible for the study, enrolled and completing 24-hour polysomnography (PSG) monitoring and sleep questionnaires on the hospital ward. *^a^*Enrolment of eligible patients was limited by the availability of only one PSG and one researcher for the entire 24-hour recording period.

During the study twenty-four hour PSG data were analysed for 53 patients and 47 patients were followed up on the ward. Of 266 eligible patients 57 were enrolled; of those not enrolled 74 declined participation and 135 were transferred to the ward before they could be invited to participate. Two patients requested removal of PSG after recording began, palliation was initiated for another and data for another was unable to be analysed. Figure 1 provides a flow diagram of the number of eligible patients and the number invited to participate and enrolled. The characteristics of patients enrolled in the study are provided in Table 1. Admission diagnoses were mostly non-operative (66%), mean APACHE II score was 18.70 (SD: 8.23) and the mean SOFA score was 4.04 (SD: 2.53). Patients were interactive and calm (mean Vancouver Interaction Scale (VICS) [22] score: 27.06 (SD: 3.80) (equivalent to 0 or -1 on the Richmond Agitation Sedation Scale (RASS) [23]).

**Table 1:** Patient characteristics

**Characteristics**	**n = 53**
Females, n (%)	17 (32)
Diagnosis, n (%)	
Operative	18 (34)
Non-operative	35 (66)
Age, mean (SD)*^a^*, y	60.13 (20.02)
APACHE*^b^*II score, mean (SD)	18.70 (8.23)
SOFA*^c^*score, mean (SD)	4.04 (2.53)
Sedation level on enrolment (VICS*^d^*), mean (SD)	
Interaction score	27.06 (3.80)
Calmness score	29.00 (2.70)
BMI*^e^*in kg/m^2^, mean (SD)	24.50 (4.90)
Duration of ventilation, median (IQR*^f^*), d	6.00 (1.67-21.50)
Length of ICU stay, median (IQR), d	12.00 (6.00-26.00)
Length of hospital, median (IQR), d	29.00 (17.50-49.50)
ICU admission day on which sleep monitoring occurred, median (IQR), d	5.00 (2.50-11.00)
Patients receiving mechanical ventilation during PSG, n (%)	28 (54)
Patients receiving an opioid, benzodiazepine or propofol, n (%)	43 (81)
Patients administered opioid and benzodiazepine medications*^g^*, n (%)	16 (30)
Patients administered benzodiazepine/propofol, n (%)	28 (53)
Patients administered opioids, n (%)	32 (60)

*^a^*SD, standard deviation; *^b^*APACHE, Acute Physiology and Chronic Health Evaluation; *^c^*SOFA, Sequential Organ Failure Assessment; ^*d*^VICS, Vancouver Interaction Calmness Scale (I = interaction score 5-30, higher scores are desirable, C = calmness score 5-30, higher scores are desirable), *^e^*BMI, body mass index; *^f^*IQR, interquartile range; *^g^*including propofol.

Twenty-one percent of patients reported a pre-hospital ISI score of ≥15, indicating moderate to severe clinical insomnia, however, median pre-hospitalisation sleep quality on the SICQ was 8.00 out of 10.00. There was no difference between the mean RCSQ score in ICU and on the ward (*P *= 0.61). Noise was rated the highest sleep-disturbing factor (Table 2).

**Table 2:** Sleep outcomes: subjective reports

**Outcomes**	**Patients (n = 48)**
ISI*^a^*score, median (IQR*^b^*)	6.00 (1.00-13.75)
ISI score ≥15, n (%)	10 (21)
Sleep quality pre-hospitalisation, median (IQR), 1-10	8.00 (5.00-9.00)
	**Patients (n = 40)**
Total RCSQ*^c^*score in ICU, median (IQR), mm	57.50 (32.00-70.00)
	**Patients (n = 45)**
Total RCSQ score in ward, median (IQR), mm	57.40 (36.70-74.40)*^d^*
	**Patients (n = 43)**
Sleep disturbing factors (SICQ*^e^*), mean (SD*^f^*), 1-10	
Noise	5.70 (2.75)
Nursing interventions	5.05 (2.44)
Light	5.15 (2.61)
Diagnostic testing	4.49 (2.67)
Vital signs	4.25 (2.12)
Blood samples	4.01 (2.20)
Administration of medications	3.84 (2.12)

*^a^*ISI, Insomnia Severity Index; *^b^*IQR, interquartile range = standard deviation; *^c^*RCSQ, Richards Campbell Sleep Questionnaire; *^d^*related samples Wilcoxon signed ranks test comparing total RCSQ ICU and ward scores *P *= 0.61, *^e^*SICQ, Sleep in Intensive Care Questionnaire; *^f ^*SD, standard deviation.

### *Sleep, sound and light*

**Figure 2:**
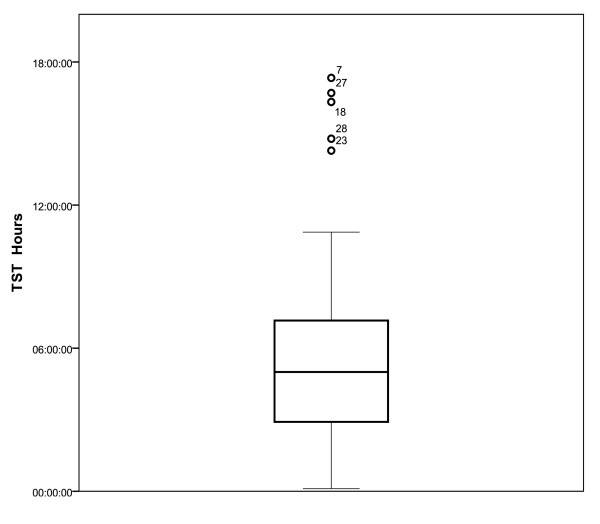
****Boxplot of total sleep time (TST) (hh:mm:ss) for the sample****. There was a large variation the TST for the sample.

Median TST was five hours (Figure 2). The median duration of sleep without waking was 00:03 (hh:mm). Unusual sleep stage transitions were noted (for example progression from stage 1 to REM sleep). Daytime sleep comprised 41% of TST and EEG delta wave activity was observed when patients were interactive and apparently awake. The majority of sleep was stages 1 and 2 (19 and 73 %, respectively). SW and REM sleep was scant (median (IQR) 0 (0 to 1) and 0 (0 to 6) respectively). Arousals were frequent (median arousal index: 27) (Table 3). Interrater reliability of the sleep technologists' PSG analysis for sleep/awake was 0.58 to 0.68 (Table 4).

**Table 3:** Sleep outcomes: PSG-derived data, sleep time and stages

**Outcomes**	**(n = 53)**
Duration of PSG recording, median (IQR), hh:mm	24:00 (23:37-24:00)
TST*^a^*, median (IQR*^b^*), hh:mm	05:00 (02:52-07:14)
Duration of sleep without waking, median (IQR), hh:mm	00:03 (00:02-00:05)
Number of sleep periods, median (IQR)	38.00 (19.00-56.50)
Sleep during daytime hours, median (IQR), %	41 (24-55)
Stage 1, median (IQR), hh:mm	01:00 (00:22-01:31)
Stage 1, median (IQR), %	19 (8-31)
Stage 2, median (IQR), hh:mm	03:03 (01:36-05:19)
Stage 2, median (IQR), %	73 (58-87)
Slow wave sleep, median (IQR), hh:mm	00:00 (00:00-00:04)*^c^*
Slow wave sleep, median (IQR), %	0 (0-1)*^c^*
REM*^d^*, median (IQR), hh:mm	00:00 (00:00-00:21)*^e^*
REM, median (IQR), %	0 (0-6)*^e^*
Arousals, median (IQR), No. per hr	27.00 (14.00-37.50)

*^a^*TST, total sleep time; *^b^*IQR, interquartile range; *^c^*range = 00:00-01:58, 0-39%; *^d^*REM, rapid eye movement; *^e^*range = 00:00-01:52, 0-22%.

**Table 4:** Interrater reliability (Cohen's Kappa statistic) for Rechtschaffen and Kales scoring for sleep technologists (16 recordings)

**Sleep stage/state**	**Technologists one and two, 18,644 epochs (95% CI*^a^*)**	**Technologists two and three, 25,908 epochs (95% CI)**
Stage 1	0.12 (0.10-0.13)	0.08 (0.06-0.10)
Stage 2	0.58 (0.46-0.72)	0.55 (0.54-0.56)
Stage 3/4	0.76 (0.70-0.82)	0.20 (0.14-0.23)
REM*^b^*	0.44 (0.39-0.49)	0.41 (0.36-0.44)
Sleep/wake	0.68 (0.65-0.69)	0.58 (0.55-0.59)

*^a^*CI, confidence interval; *^b^*REM, rapid eye movement.

Sound pressure levels were high (mean Leq 53.95 (SD: 2.33) dB(A) during the day and 50.20 (SD: 3.21) dB(A) at night). There were a median 416/h sound peaks >80 dB(C) during the day and 90/h at night. The correlation between arousal indices and number of sound peaks >80 dB(A) was weak during the day (r = 0.13) and night (r = 0.19); neither was statistically significant. Median illuminance levels were lower at night (1.74 lux) than during the day (74.20 lux). The average number of care events was 1.74/h; the lowest was between 0200 and 0500 hours (<1.00/h). No patients wore ear plugs or eye shades during sleep monitoring.

### *Sleep mechanical ventilation and medications*

Pressure support was the ventilation mode used for 26 patients (six patients received pressure control ventilation (PCV) for periods during pressure support), one patient received synchronised intermittent ventilation (SIMV) and another patient received PCV. Twelve patients were extubated (that is, had their endotracheal tube removed) during PSG monitoring. The median TST of patients who received mechanical ventilation during PSG recording and those who did not was 05:14 (IQR: 03:36 to 07:57) vs. 03:57 (IQR: 01:39 vs. 06:47; *P *= 0.049) and median arousal indices (20.50 IQR: 11.50 to 32.5 vs. 29.50 IQR: 22.00 to 40.87, *P *= 0.018). The differences for other sleep parameters did not reach statistical significance for patients who received mechanical ventilation during PSG recording and those who did not (for example, stage 1: 22.87 (24.13) vs. 27.54 (19.85) %, stage 2: 69.99 (24.02) vs. 67.30 (20.17) % and REM: 4.47 (5.90) vs. 2.38 (4.91) %).

The mean equivalent dose of morphine was 12.46 (SD: 24.42) mcg/kg/h and equivalent dose of midazolam was 2.26 (SD: 11.60) mcg/kg/h. Six patients received temazepam (four received 20mg and two 10mg) for night sedation. There was a difference in the median arousal indices for patients who received benzodiazepine medication/propofol and those who did not (22.00 IQR: 11.25 to 31.75 vs. 30.00 IQR: 20.75 to 40.75; *P *= 0.019). The differences for other sleep parameters did not reach statistical significance for patients who received benzodiazepine medication/propofol and those who did not (for example, stage 1: 26.41 (20.74) vs. 23.71 (23.75) %, stage 2: 67.42 (19.38) vs. 69.97 (24.74) % and REM: 2.85 (4.76) vs. 4.14 (6.16) %).

## Discussion

This study provides a current characterisation of the quality and quantity of sleep in intensive care patients, together with factors that potentially affect the sleep of patients in ICU. Sleep quality was poor; over 90% of patients' sleep was stage 1 and 2 and TST was below that experienced by healthy adults. These results indicate that there has been no improvement since studies published between 1976 and 2003 [3,5,24-26], despite improvements in ICU design, technology and health care personnel training. Sleep fragmentation and unconventional architecture was evident with multiple short, non-contiguous sleep periods and numerous arousals. Arousals were frequent, as well as non-sequential stage changes [4,27]. A large percentage of TST was during daytime hours

Sound levels were elevated and exceeded World Health Organization (WHO) standards for hospitals, that is Leq should not >35 dB(A) in patient areas [28]. In our study, Leq was >49 dB(A). Continuous equivalent sound pressure levels were 10 dB(A) lower than in many studies in ICU [5,29,30] but similar to others [26,31]. The frequency of sound peaks >80 dB(C) was notable (range: 31 to 1,436/h) and high in comparison with other studies. Kahn *et al*. [32] reported a mean of 56/h before a noise reduction program and 40/h afterwards, while the mean reported by Meyer *et al*. [33] was 60/h. It is somewhat surprising that there was no correlation between arousal indices and the number of sound peaks >80 dB(A), although it is possible that patients had become accustomed to high sound levels. Illuminance levels were appropriate at night (median <2 lux). However, daytime illuminance levels (74 lux) may have been too low to encourage a 24-hour circadian rhythm. Study of endogenous melatonin secretion in healthy participants indicates that illuminance levels of <100 lux may not be sufficiently bright to suppress melatonin secretion in some individuals [34]. Thus patients in our study may have had high melatonin levels during the day that contributed to the proportion of daytime sleep. The mean number of treatment/care events was 40 (1.7/h). This contrasts with hourly rates reported by others (for example 3, 7 and 6/h) [6,25,26]. Under-recording by the bedside nurses may have occurred in our study. While an attempt was made to record care events/treatment in a synchronous manner, this proved difficult in practice. Hence we are unable to draw conclusions about the relationship between such events and arousals.

Medications administered and considered to potentially affect sleep noted during PSG recording were mainly opioids and sedative medications. A large proportion (60%) of patients received opioid medications, potentially affecting their sleep. SWS has been found to be reduced by opioids, with a concomitant increase in stage 2 sleep [35]. A large percentage of patients in our study received benzodiazepines or propofol (53%) (however, only lightly sedated patients were enrolled; the mean VICS score was 27.06 (SD: 3.80)). This may also have contributed to the high proportion of stage 2 and lack of REM sleep [36]. However, it is notable that patients who received benzodiazepine medication or propofol had fewer arousals than patients who did not. Despite the apparent suppression of SWS and REM, sleep may have been more consolidated when sedative medications were administered. The somewhat surprising findings that TST was higher and there were fewer arousal indices in patients who received mechanical ventilation during PSG recording may be partly attributable to the larger sedative medication doses they received (mean: 3.49 (SD: 15.64) vs. 0.40 (SD: 0.75) mcg/kg/h).

Patients' self-reports of sleep quality in ICU were poor but comparable to previous studies. The mean RCSQ score from patients ready for transfer to the ward in the same ICU (n = 222) was 47.18 mm [37]. In a study investigating the utility of the RCSQ and concordance of nurse and patient sleep assessment in ICU, the mean RCSQ score was 45.50 mm [38].

Patient perceptions of sources of sleep disruption were similar to previous studies. Noise was rated the most disruptive, as has been reported elsewhere [26]. In the development of the SICQ, ratings of disruptive activities were lower and noise (4.5) was less disruptive than vital signs (5.5) and phlebotomy (5.5) [10]. However, the SICQ was first reported in 1999; non-invasive vital signs monitoring and blood sampling in the current study would be expected to be less disruptive than techniques in use in the 1990s.

### *Study strengths and limitations*

This study is the largest of its kind to be conducted using PSG with simultaneous data collection for factors known to affect sleep in ICU and is the first to present data from the Australian context. In addition, other investigators have rarely collected data on the patients' perception of sleep quality and potential sleep-disrupting factors in conjunction with PSG recording. This subjective information is vital in corroborating objective data, particularly as sleep is a subjective experience.

A limitation that became evident during the study was the difficulty in interpreting the PSG data using conventional R and K analysis. One patient's EEG waveform was affected with 'alpha intrusion' (alpha wave activity superimposed on delta waves), which made analysis impossible (the likely explanation being the administration of antipsychotic medications after enrolment/PSG recording began). Observation of EEG delta wave activity in some patients who appeared to be awake has been noted in other studies [4,5,39]. Challenges in scoring ICU patients' sleep data were recently reported by Drouot *et al*. [39] in their analysis of PSG data from two studies conducted previously. The investigators noted the presence of EEG delta wave activity during apparent wakefulness (the presence of EMG, EOG and limb activity) and a lack of K complexes and sleep spindles preventing classification of stage 2. However, interrater and intrarater scorer reliability checks were not performed in that study.

Another limitation is the effect of benzodiazepine medications on the interpretation of sleep parameters. It is known that benzodiazepine medication increases EEG beta wave activity and reduces EEG delta wave activity [40]. Benzodiazepine medication also increases EEG spindle activity [41] (though this appears not to have been the case in our study). The combined effect of the opioids and sedative medication on EEG activity likely affected the results. However, since these medications are essential adjuncts in the treatment and comfort of many ICU patients, excluding patients who received them would have severely limited recruitment and the generalisability of our results. Other limitations were the presence of factors such as the use of different modes of mechanical ventilation and health conditions such as systemic inflammatory response, but such factors are common in ICU patients and their exclusion would also limit the applicability of the results to the ICU patient population.

Patient enrolment was limited by a number of factors. The availability of PSG monitoring equipment and the principal researcher were the primary factors for patients being discharged before they were approached to participate. For this study, we had access to only two people trained in sleep recordings (RE and MF) and one portable sleep-monitoring device, creating practical limitations to the number and frequency of studies performed. In addition, there was often limited opportunity in which patients were cognitively able to agree to participate before they were discharged to the hospital ward. A large proportion of patients approached about the study declined to participate; many considered the application of monitoring as a set-back in their condition regardless of the reason for it. This may limit the generalisability of the results to a sub-set of patients treated in the study ICU.

Interrater reliability of the R and K analysis was moderate, 0.56 for sleep technologists one and two and 0.51 for sleep technologists two and three but lower than Kappa values for sleep technologists in sleep investigation units (for example 0.72 [42]). Interrater reliability for each stage was also lower than in sleep laboratory studies. The reliability of PSG analysis in ICU patients has been infrequently reported. In one study of critically ill, non-ventilated trauma patients' night-time sleep, interrater reliability for one overnight recording, was reported as Kappa = 0.82 [43]. However, in a comparison by Ambrogio *et al*. of four methods of analysing ICU patients' PSG data, interrater reliability was considerably lower than in the present study (Kappa = 0.19) [44].

## Conclusions

The quantity and quality of patients' sleep while in this ICU using both objective and subjective assessment methods were found to be poor. Given the similarity between the sleep outcomes and prevalence of potential sleep-disturbing factors in our study and previous studies, sleep disruption clearly remains a substantial problem for many ICU patients. Importantly, the current study has added to evolving knowledge of the challenges of using PSG in ICU and analysing the data. An alternative objective sleep assessment method is required for ICU patients in order to further our understanding of sleep disruption in this vulnerable patient population and to test clinical interventions for their well-being and recovery.

## Key messages

·Sleep in ICU patients is highly fragmented with concomitant deficiencies in SW and REM sleep.

·Use of PSG in ICU patients and analysing the data are challenging.

·There is a need to develop alternative methods to conventional PSG staging to measure sleep in ICU patients and develop interventions which will improve sleep.

## Abbreviations

APACHE, Acute Physiology and Chronic Health Evaluation; ECG, electrocardiograph; EEG, electroencephalograph; EMG, electromyograph; EOG, electrooculograph; ICU, intensive care unit; ISI, Insomnia Severity Index; Leq, equivalent sound pressure levels; Lpeak, peak sound pressure levels; PCV, pressure control ventilation; PSG, polysomnography; R and K, Rechtschaffen and Kales; RASS, Richmond Agitation Sedation Scale; RCSQ, Richards-Campbell Sleep Questionnaire; REM, rapid eye movement; RN, registered nurse; SICQ, Sleep in Intensive Care Questionnaire; SIMV, synchronised intermittent ventilation; SOFA, Sequential Organ Failure Assessment; SWS, slow wave sleep; TST, total sleep time; VAS, visual analogue scale; VICS, Vancouver Interaction Scale.

## Competing interests

The authors declare that they have no competing interests.

## Authors' contributions

RE conceived and developed the protocol and design, acquired and analysed the data, wrote the manuscript, SM and PC supervised RE, assisted with the development of the design, interpretation of the data and writing the manuscript and MF assisted with protocol development, data acquisition and writing the manuscript. All the authors have read and approved the manuscript for publication.
